# Neural Response Attenuates with Decreasing Inter-Onset Intervals Between Sounds in a Natural Soundscape

**DOI:** 10.1523/ENEURO.0210-25.2025

**Published:** 2025-10-17

**Authors:** Thorge Haupt, Marc Rosenkranz, Martin G. Bleichner

**Affiliations:** ^1^Neurophysiology of Everyday Life Group, Department of Psychology, Carl von Ossietzky Universität Oldenburg, Oldenburg 26129, Germany; ^2^Research Center for Neurosensory Science, Carl von Ossietzky Universität Oldenburg, Oldenburg 26129, Germany

**Keywords:** auditory evoked potentials, natural soundscape, neural attenuation, temporal response functions

## Abstract

Sensory attenuation of auditory evoked potentials (AEPs), particularly N1 and P2 components, has been widely demonstrated in response to simple, repetitive stimuli sequences of isolated synthetic sounds. It remains unclear, however, whether these effects generalize to complex soundscapes where temporal and acoustic features vary more broadly and dynamically. In this study, we investigated whether the inter-onset interval (IOI), the time between successive sound events, modulates AEP amplitudes in a complex auditory scene. We derived acoustic onsets from a naturalistic soundscape and applied temporal response function (TRF) analysis to electroencephalography data recorded from normal hearing human listeners (*N* = 22, 16 females, 6 males). Our results showed that shorter IOIs are associated with attenuated N1 and P2 amplitudes, replicating classical adaptation effects in a naturalistic soundscape. These effects remained stable when controlling for other acoustic features such as intensity and envelope sharpness and across different TRF model specifications. Integrating IOI information into predictive modeling revealed that neural dynamics were captured more effectively than simpler onset models when training data were matched. These findings highlight the brain’s sensitivity to temporal structure even in highly variable auditory environments, and show that classical lab findings generalize to naturalistic soundscapes. Our results underscore the need to include temporal features alongside acoustic ones in models of real-world auditory processing.

## Significance Statement

Employing automatic onset detection in a complex, ecologically valid soundscape, we enable fine-grained analysis of temporal auditory processing. Specifically, we find that neural responses (i.e., the N1 and P2 components) to sound events are attenuated when inter-onset intervals are short, replicating classic attenuation effects within a naturalistic soundscape. These findings demonstrate that temporal sensitivity in auditory processing persists even in the presence of substantial acoustic variability, which is characteristic of real-world settings.

## Introduction

Non-invasive neuroimaging tools, such as electroencephalography (EEG), have been invaluable in unraveling the neural underpinnings of auditory perception (Alain and Winkler, 2012; Gutschalk and Dykstra, 2014; Lee et al., 2014; Rahman et al., 2020). Many of the mechanisms uncovered using EEG have relied on highly controlled, low-complexity stimuli (Crosse et al., 2016; Schutz and Gillard, 2020). Often, unnatural and repetitive, click-like tones have been used, reducing experimental investigation to changes along a single stimulus dimension, such as intensity (López-Caballero et al., 2023), frequency (Herrmann et al., 2013, 2014), or inter-stimulus interval (ISI; Zacharias et al., 2012). An emerging question is whether established neural mechanisms, such as the attenuation of auditory evoked potentials (AEP), can be applied to understand human perception in response to complex and naturalistic soundscapes, where many of the investigated factors occur simultaneously. A well-documented finding is that the amplitude and latency of an AEP in response to a sound are dependent on the characteristics of the preceding sound, as well as the context. Studies have shown that repeated presentations of tones modulate AEP components associated with acoustic processing, particularly the N1. It has been shown that the N1 amplitude is reduced by a preceding tone for up to 10 s (Wang et al., 2008; López-Caballero et al., 2023). Furthermore, it has been shown that the N1 amplitude scales non-linearly with ISI (Zacharias et al., 2012). While the peak modulation has been observed reliably, the exact neural mechanisms driving this attenuation remain debated (Näätänen and Picton, 1987; May and Tiitinen, 2010). Taken together, these findings demonstrate that the N1 component is sensitive to specific stimulus properties, such as temporal spacing. Importantly, changes in these acoustic properties lead to predictable patterns of modulation in amplitude and latency of the neural response.

Recent advances in data analysis and experimental designs have made it feasible to study auditory processing in response to more naturalistic sound environments (Lalor et al., 2009; Holdgraf et al., 2017; Crosse et al., 2021; Brodbeck et al., 2023) and situations (Ladouce et al., 2021; Rosenkranz et al., 2023, 2024). The trend toward using more naturalistic stimuli is particularly notable in language research, where studies have gradually moved from presenting isolated words and phonemes (Lutzenberger et al., 1994; Näätänen, 2001) to sentences (Desai et al., 2021), continuous speech (Howard and Poeppel, 2010; Ding and Simon, 2014), and, ultimately, naturally recorded speech (Agmon et al., 2023). This shift toward naturalistic stimuli provides new insights into auditory processing and raises the question of how far results based on experiments using isolated tones generalize to real-world soundscapes (Schutz and Gillard, 2020; Hamilton et al., 2021; Vallet and van Wassenhove, 2023).

Temporal response functions (TRFs) allow the study of neural responses to continuous acoustic stimuli (Holdgraf et al., 2017; Kriegeskorte and Douglas, 2019; Crosse et al., 2021), allowing us to investigate whether auditory mechanisms derived from isolated tones extend to real-life sounds. The benefit of these models is that they are straightforward to interpret and allow for the comparison of multiple models (Crosse et al., 2016). However, many standard TRF implementations assume, either explicitly or implicitly, that neural responses to repeated instances of a given feature type, such as peaks in the speech envelope, are uniform. This assumption oversimplifies neural dynamics, as accumulating evidence suggests that brain responses to acoustic features are often non-linear and context-dependent (Stam, 2005; Wang et al., 2008; Buzsáki and Mizuseki, 2014; Herrmann et al., 2016). This raises a critical question: How can prediction-based models account for the non-linear and temporally dynamic nature of auditory processing?

Drennan and Lalor (2019) approached the modeling of non-linear neural dynamics by partitioning the acoustic speech envelope into discrete amplitude-based bins. This enabled a more precise characterization of intensity-dependent neural responses to continuous auditory stimuli.

We aim to utilize the approach of Drennan and Lalor (2019) and investigate whether the influence of inter-onset interval (IOI; i.e., the temporal distance between two onsets) on neural response amplitude, which was previously observed using simple, isolated stimuli, can be extended to complex, naturalistic soundscapes. The investigation of the effect of IOI on neural response amplitude is non-trivial, since sound events rarely occur at a steady rhythm and differ widely in their acoustic properties. Generalizing this relationship to real-world auditory input would provide a framework for understanding brain dynamics in a more ecologically valid setting. Specifically, we test whether the amplitude of the neural response to a sound onset depends on the duration of the interval preceding it, even in the presence of continuous, naturalistic auditory input.

## Method

### Data set

The current study uses an existing data set by Rosenkranz et al. (2023), where they investigated the effect of attentional modulation on auditory perception during a complex audio-visual motor task. Specifically, the soundscape was created to simulate sounds encountered in an operating room to determine the neural response to different types of relevant and irrelevant sounds depending on the attentional instructions. In this dataset, 22 healthy, right-handed adults (age range: 20–30; 6 males, 16 females) were recruited through an online announcement. All participants provided informed consent and received monetary compensation. The sample size was determined based on previous studies investigating similar neural markers in natural settings (Scanlon et al., 2019; Hölle et al., 2021). Eligibility criteria included normal or corrected-to-normal vision, self-reported normal hearing, absence of psychological or neurological conditions, right-handedness, and compliance with COVID-19 hygiene regulations in place at the time of data collection. Two participants were excluded from analysis: one due to poor EEG data quality and another for not following task instructions. Therefore, the final analyzed sample comprised 20 participants (14 females, 6 males).

#### Code accessibility

The code described in the paper is freely available online at https://github.com/ThorgeHaupt/Attenuation.git. The analyzed dataset can be found under https://zenodo.org/records/7147701. A Dell Precision 3,650 Tower running Microsoft Windows 10 Education was used.

### Task

The goal of the original study was to investigate attentional effects in a surgical workplace scenario. For this, participants had to perform a complex visual motor task that comprised playing three-dimensional Tetris. In addition to the standard Tetris rules, vocal instructions occasionally told the participants specific locations where to place the blocks. Besides the vocal instructions, participants had to respond to tones. There were two conditions in which the participants had to respond to different tones. Specifically, participants were instructed to respond either to a distinct alarm tone (narrow attentional scope) or a less distinct beep tone (wide attentional scope). Both tones occurred within each condition, with only the target instructions differing between conditions. They are, however, not relevant for the current analysis.

### Soundscape

The soundscape was designed to mimic an operating room, specifically geared toward a surgeon’s perspective. The acoustic environment consisted of speech sounds and environmental sounds (e.g., clattering of tools, footsteps, conversations). The sounds were either vocal instructions on where to place the next block or conversation snippets from a podcast. In total, each participant had to comply with the vocal instructions 12 times, where they were told to “Place the next stone in the [upper∖lower left∖right] corner.” Importantly, the instructions were played randomly and never consecutively repeated. The conversation snippets were taken from a podcast and also placed randomly, but in semantically coherent order. The content of the conversation snippets was irrelevant to the experiment. In total, 48 snippets were played and lasted roughly 3.5( ± 1.5) s. The total soundscape was played for roughly 16 min per condition, totaling 32 min of recorded data on average.

**Figure 1.**
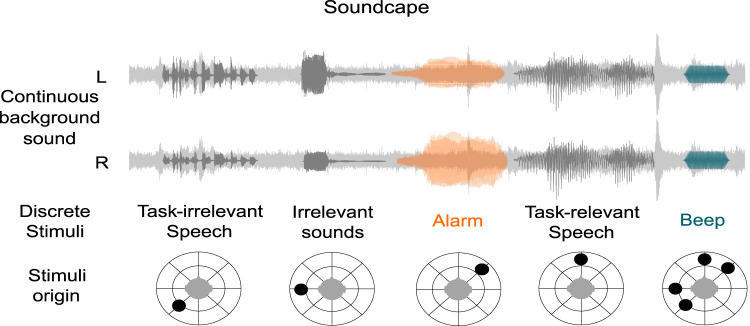
Illustration of the experimental soundscape presented binaurally via headphones (left and right channel shown separately). Light gray indicates continuous surgical background noise. Dark gray marks task-irrelevant sound events, including vocal instructions and irrelevant speech snippets. Orange indicates the alarm tone relevant in the narrow-attention condition, while dark green marks the beep tone relevant in the wide-attention condition. The circular schematics below each discrete stimulus illustrate their spatial positions, manipulated using head-related transfer functions. Adapted from Investigating the attentional focus to workplace-related soundscapes in a complex audio-visual motor task using EEG by Rosenkranz et al. (2023). Licensed under CCBY.

Besides speech segments, the soundscape also contained hospital sounds of people moving around and air conditioning. Furthermore, there were three different tones inserted: alarm, beep, and irrelevant. The alarm and irrelevant sound were 200 ms, and the beep tone was 60 ms long. Each tone was played 48 times and was also randomly placed into the soundscape. Importantly, the experimentally relevant alarm tone was always played from the same direction, whereas the beep was played from multiple directions. Both tones (alarm and beep) were always presented in both experimental conditions, differing only in which tone participants were instructed to respond to. The timing of tone presentations within the soundscape was randomized individually for each participant. When randomization resulted in the beep tone overlapping with other tones (e.g., beep and alarm, or beep and irrelevant sounds), the overlapping sounds were returned to the stimulus pool and presented again at a new randomized time. This was done to obtain 48 non-overlapping trials to unbias the following EEG analysis of the tones. Consequently, slight variations in total condition duration occurred across participants. However, each participant consistently received the full stimulus set: 48 vocal instructions (2–3 s each), 48 conversation snippets (mean duration 3.5±1.5 s each), and 144 total tone presentations [48 each: alarm (200 ms), beep (60 ms), irrelevant (200 ms)], embedded within continuous environmental background sounds (Fig. 1). Including brief silent intervals between stimuli and background environmental sounds, this procedure yielded an average soundscape duration of approximately 18 min per condition, totaling around 36 min across both conditions. Although durations varied slightly due to randomization, these differences were marginal and not expected to introduce systematic effects on time-on-task analyses. For additional clarity, a schematic timeline illustrating stimulus sequencing and timing has been added (adapted from Rosenkranz et al., 2023).

All sounds included in the soundscape were processed in MATLAB, such that the root-mean-squared (RMS) was consistent across them. Accounting for differences in loudness was done by adjusting the loudness through sound-specific gain parameters. Lastly, using the head-related impulse function, tones were spatially separated. The experimental audio stimuli were sampled at 44.1 kHz. All recorded data streams were synchronized via the Lab Recorder software, utilizing the lab streaming layer for integration. Participants provided informed consent after being briefed on the procedure. For more details, see the original paper (Rosenkranz et al., 2023).

### EEG measurement

Participants were fitted with 24 Ag/AgCl passive electrodes positioned according to the 10–20 international system (EasyCap GmbH) for EEG recording. Data collection was performed using a wireless SMARTING system (mBrainTrain), with signals referenced to Fz and grounded to AFz. Sampling occurred at a frequency of 500 Hz, and electrode impedance was kept below 20 Ω prior to recording.

### Preprocessing of EEG data

EEG preprocessing was conducted using MATLAB (version 2021a, MathWorks) with the EEGLab plugin and supplementary custom scripts. Artifact detection was performed using independent component analysis (ICA). To optimize ICA weight estimation, separate preprocessing steps were employed, as recommended by Winkler et al. (2015). This preprocessing pipeline was solely designed for ICA computation and thus did not influence the data ultimately used for analysis. After deriving the ICA weights, they were applied to the unprocessed raw data.

Initially, data from both experimental conditions were combined for each participant. The combined data was resampled to 250 Hz and subjected to a series of filters, starting with a high-pass filter (cutoff: 1 Hz, order: 568) followed by a low-pass filter (cutoff: 42 Hz, order: 128). These cutoff frequencies were chosen to mitigate drifts and line noise, facilitating optimal ICA weight estimation (Winkler et al., 2015). Channels exhibiting poor signal quality were removed using the clean_channels function. The data was segmented into 1-s epochs, converted to double-precision format, and artifactual trials were removed using the pop_jointprob function with a threshold of three standard deviations.

ICA was executed using the pop_runica function with the extended ICA algorithm. The resulting ICA weights were then reapplied to the raw, unfiltered data from each experimental condition. Automatic classification of ICA components as muscle, eye, heart, line noise, or channel noise artifacts was performed using the pop_icaflag function, with a pre-defined probability threshold ([0.7, 1; 0.7, 1; 0.6, 1; 0.7, 1; 0.7, 1]). Here the conservative rejection thresholds were chosen to account for the button presses through the conditions.

Following artifact removal, the raw data underwent a second round of filtering, this time with modified parameters. A low-pass filter was applied first (cutoff: 20 Hz, order: 100), followed by resampling to 100 Hz and high-pass filtering (cutoff: 0.3 Hz, order: 518). The reduced low-pass filter order minimized artifacts associated with steep roll-offs, as recommended by Crosse et al. (2021). The frequency band was restricted to [0.3, 20] Hz, aligning with findings from speech-tracking studies highlighting the dominance of auditory processing in lower frequency ranges (Di Liberto et al., 2015; Crosse et al., 2016). Finally, the data was rereferenced to the mastoids (TP9/TP10).

### Temporal response function

The neural time series were analyzed using the mTRF toolbox (Crosse et al., 2016) in MATLAB. This toolbox estimates weights that relate neural responses to stimulus features through convolution. The neural response, ***r***(*t*, *c*), is modeled as the convolution of channel-specific weights (TRF), *ω*(*τ*, *c*), with the stimulus features shifted by a time lag, *τ*, plus a residual term, ε(t,c):

$$r(t,c)=\sum_{\tau }\omega (\tau ,c)s(t-\tau )+\varepsilon (t,c).$$

Here, *t* and *c* denote time points and channel indices, respectively. This approach captures the delayed nature of neural responses to stimuli. The resulting weights were analyzed for morphology, topography, model performance, multivariate modeling, and cross-prediction.

The TRF is determined by minimizing the mean squared error (MSE) between observed and predicted neural responses:

$$\min_{\hat{r}}\sum_{t}[r(t,c)-\hat{r}(t,c){]}^{2}.$$

The optimal weights, ***w***, are computed using the formula:

$$w=(S^{⊤}S{)}^{-1}S^{⊤}r.$$

Here, *S* is the design matrix containing the stimulus features across time lags. Its dimensionality is determined by the number of features and lags. Zero-padding was applied at non-zero lags to maintain causality (Mesgarani et al., 2009). The operation $S^{⊤}r$ represents the inner product between stimulus and neural time series, while $(S^{⊤}S{)}^{-1}$ accounts for stimulus autocorrelation.

The dimensions of the resulting model weights are determined by the number of features, the time window of integration, and the number of channels. For instance, dividing the soundscape into eight different bins of IOI intervals yields a model with the dimensionality of 8 × 61 × 22.

### Model training

To train the model, the data was partitioned into six segments, where five served for training and one segment served as the held-out segment for testing. Within the five training segments, cross-validation was applied to derive the optimal lambda for regularization. The resulting model was used to predict the data of the test segment and correlated to the actual neural data. The correlation is the performance marker and is indicative of the prediction accuracy of the model. This approach was consistently applied over all analyses unless stated otherwise. A typical time lag window of [−100, 500] ms was used unless stated otherwise. Cross-validation included a lambda parameter search over values ranging from 10^−4^ to 10^4^ in linear steps of 10.

To investigate the role of training data, we increased the available data and contrasted the bin IOS models against the single onset vector. First, we merged the datasets of the two conditions per participant. We then divided the concatenated data into 12 segments, each serving as a test set once.

### Analyses

#### Features

##### Onsets

We are interested in the effect of the IOI of two consecutive sounds on the neural response. Unlike previous studies that investigated this effect with pure tones, we are interested here in whether we can replicate the effect in complex soundscapes. For this, we needed to identify sound onsets in the continuous soundscape. To obtain onsets, we used peak detection of acoustic novelty functions (Müller, 2021), which are defined by changes in the energy, spectral flux, and phase changes of the signal, respectively. Given that onset detection was performed on the raw audio signal, no distinction was made between specific sound categories (vocal instructions, conversation snippets, tones, or background noise). Therefore, all detected acoustic onsets were treated equally and weighted identically in the subsequent TRF analyses. This approach intentionally disregards semantic or categorical aspects of the soundscape to focus purely on acoustic temporal structure. For a detailed discussion of how using purely acoustic rather than content-informed onsets impacts the estimation and interpretation of neural responses, see Haupt et al. (2024).

##### Energy novelty

The underlying assumption of the first novelty function is that sound event onset leads to changes in the energy of the signal (*x*). To obtain this representation, the raw signal was squared, and a continuous measure of local energy was obtained by convolving it with a Hann windowing function. Next, the signal was downsampled to the EEG sampling rate at 100 Hz. Given that sound perception of different intensities is logarithmic in humans, we applied a logarithmic compression log(1+γ*x), where the compression is controlled by *γ* = 10. Finally, the rate of change of the signal was obtained by taking the derivative and half-wave rectifying it.

##### Spectral novelty

The second novelty function we derived was the spectral flux. Instead of depicting changes in the broadband signal, where overlapping sounds could mask each other, spectral decomposition into different frequency bands can provide a more detailed account of acoustic changes in the signal. First, the signal was decomposed into its frequency components using the short-time Fourier transform (STFT). The magnitude in each frequency band was obtained by taking the absolute value and applying logarithmic compression (*γ* = 10). To determine the rate of change in each frequency band, the first derivative was taken, and the signal was half-wave rectified. At last, the signal was obtained by summing over frequency bands. Postprocessing involved removing small fluctuations by subtracting the local average of the signal. Negative values were set to zero.

##### Complex novelty

The third novelty function extends the spectral flux function by considering changes in the phase of the signal’s frequency components. To avoid chaotic noise-like phase fluctuations impairing the novelty estimate, the phase is weighted by the magnitude of the Fourier coefficient. That is, phase information becomes only relevant, given the magnitude of the Fourier coefficient. The novelty function was derived by determining the difference between the predicted and actual signal, where larger values refer to greater change. Here, the predicted signal was construed based on the assumption of local stationarity, implying that the phase and magnitude of the Fourier coefficients stay relatively constant over some time.

Similar to the spectral flux, the signal was decomposed into Fourier coefficients using the STFT, and besides the magnitude, phase values were extracted. The angle of the coefficients was derived and normalized by 2*π*. Afterwards, the rate of change was determined by taking the derivative of the phase values. The Fourier coefficient of the next frame was predicted based on the magnitude, current phase, and rate of phase change. The difference between the actual and predicted coefficient was derived, and novelty values smaller than the previous one were set to 0. The novelty function was obtained by summing over frequencies. Local averaging and half-wave rectification were applied to obtain smoother results.

##### Onset detection

Each novelty function represents a distinct measure of change in the signal, contributing unique information. To leverage the complementary representations of change, we normalized the novelty functions between 0 and 1 and averaged them together. This approach aimed to integrate the advantages of all novelty functions, capturing a more comprehensive depiction of changes in the auditory environment.

**Figure 2.**
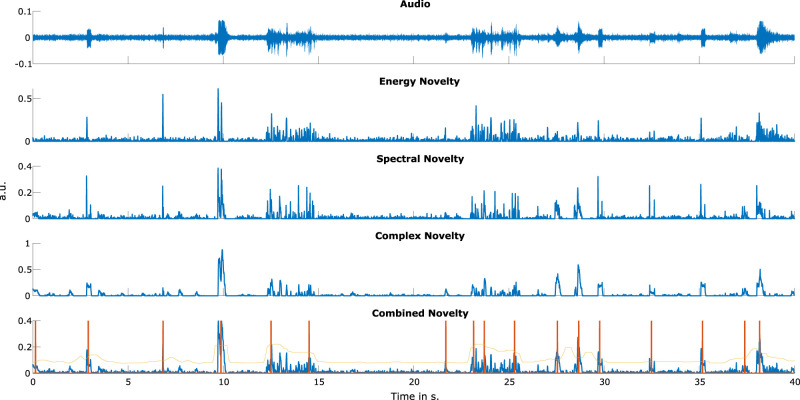
A representation of the novelty functions and the corresponding peak-picking algorithm. The top plot shows the audio signal played to Participant 1 during the narrow condition. Below are the corresponding energy, complex, and spectral novelty functions. The last plot shows the combined novelty functions in blue, the local average in yellow, and detected peaks in orange.

To detect sound event onsets, we applied an adaptive thresholding algorithm. Unlike global thresholding, which can overlook smaller, noise-like peaks, adaptive thresholding considers the local temporal structure. Specifically, we smoothed the combined novelty function using a Gaussian window (*σ* = 4) and applied an offset defined as mean(*x*) + 0.05. To further refine the signal, we employed a median filter with a window size of 1,024 samples. The resulting signal represented the local average, and a peak was only selected if the novelty exceeded the local threshold. The temporal location of each detected peak was recorded as a sound event onset. Finally, the onset information was encoded into a binary feature vector for further analysis (Fig. 2).

#### IOI analysis

For the IOI, we calculated the time interval between successive onsets. Onsets separated by more than 10 s were excluded from further analysis, based on previous research findings suggesting that neural attenuation occurs within this time frame (Zacharias et al., 2012).

**Figure 3.**
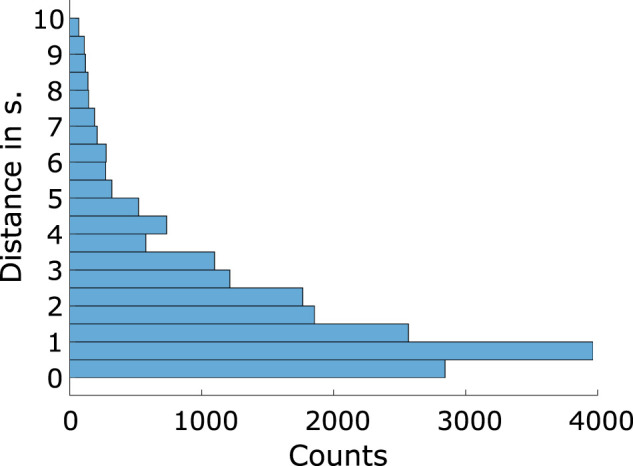
Histogram displaying the distance of onsets to the previous one over all participants.

Inspired by the method of Drennan and Lalor (2019), we applied a similar strategy based on the IOI between sound event onsets. First, we defined ranges for the IOI values and assigned each onset to its corresponding bin. To determine the bin edges, we analyzed the sample distribution of distance values (Fig. 3). This distribution of distances of the sound onsets is non-normally distributed and is skewed to the lower distance values. Here, 80% of the sound event onsets follow another sound event within 3.63 s. The binning was designed to ensure a uniform distribution of onsets across bins, meaning each bin contained an equal number of onsets. Since no prior studies had applied this approach, we experimented with different bin numbers (ranging from 2 to 8), leading to seven distinct models with varying numbers of bins. These models were then used to predict unseen data, and amplitude values were extracted.

To determine whether the peak values as a function of IOI were due to chance, we conducted a permutation analysis. Specifically, we randomly shuffled the allocation of sound onsets to their corresponding bins while preserving the overall distribution of onsets. This ensured that changes in the soundscape were still captured, but without a structured relationship to IOI. For each model, we summed the difference between each peak value, representing a rate of change score. The gradient of amplitude values over IOI for each bin model served as the aggregate score for comparison.

We applied the same analysis to 100 permuted model values, generating a distribution of 100 chance gradient values. Statistical significance was asserted at *p* < 0.05. To control for multiple comparisons, we applied a false discovery rate correction.

### Acoustic properties beyond sound event distance

To investigate whether the observed neural amplitude differences between bins could be attributed to systematic acoustic properties of the stimuli, beyond IOI, we extracted two additional acoustic markers known to influence neural response magnitude. The first was the intensity (amplitude) of the sound event. Previous research has demonstrated that sound intensity is positively non-linearly related to neural response amplitude (Adler and Adler, 1989; Drennan and Lalor, 2019; López-Caballero et al., 2023). Interestingly, López-Caballero et al. (2023) examined both intensity and ISI and found that both modulated the N1 and P2 components. Moreover, they reported a positive interaction between these factors, specifically, the modulatory effect of intensity on neural responses was more pronounced at longer ISIs, suggesting a dynamic interplay between temporal and intensity cues. The second factor was the sharpness of the envelope onset. This characteristic has posed challenges in auditory research, as sound events with slow-rising envelopes complicate the accurate determination of perceptual onset (Rosenkranz et al., 2024). In such cases, automatic onset detection may not identify the optimal alignment point, potentially resulting in temporal smearing when responses are averaged. To quantify envelope sharpness, we calculated the gradient of the waveform within the first 50 ms following onset.

For each sound event, we derived values for these two markers: amplitude and sharpness, alongside the IOI to the preceding event. To assess their respective contributions, we employed a linear mixed-effects modeling approach. Due to the low signal-to-noise ratio associated with neural responses to rapidly successive events, we did not model single-trial neural response amplitudes directly. Instead, we used IOI as the dependent variable to examine its relationship with the other acoustic predictors.

## Results

Previous research has established that for isolated tones, neural attenuation occurs when tones are played in close succession (Wang et al., 2008; Zacharias et al., 2012; López-Caballero et al., 2023). Here, we extend these findings by examining whether a similar modulation occurs in more complex, naturalistic auditory environments. Specifically, we examined whether the neural response to sound events is modulated by the IOI. To test whether accounting for varying IOI of sound onsets would show modulation of neural response in naturalistic soundscapes, we derived models by grouping sound event onsets in specific IOI ranges. We then tested whether the grouping of sound onsets into varying distance bins would also explain more neural variability.

### Modulating acoustic properties

To evaluate the potential influence of systematic acoustic differences of the sound events of the different bins, we applied a linear mixed-effects modeling approach, with participants modeled as random intercepts. Before running the model, we assessed collinearity between predictors and found significant correlations between distance and intensity (*r* = −0.15, *p* < 0.001) and between sharpness and intensity (*r* = 0.35, *p* < 0.001), suggesting moderate interdependence among these variables. The correlation could impair the estimated coefficients.

The final model included sharpness, intensity, and their interaction (sharpness * intensity) as fixed effects, with participants as random intercepts. The analysis revealed a significant main effect of intensity [*β* = −195.92, SE = 9.75, *t*(18, 327) = −20.09, *p* < 0.001], indicating that higher sound intensity was reliably associated with shorter IOIs. In contrast, the main effect of sharpness was not significant [*β* = 12.64, SE = 14.99, *t*(18, 327) = 0.84, *p* = 0.399], nor was the interaction between sharpness and intensity [*β* = 17.41, SE = 21.33, *t*(18, 327) = 0.82, *p* = 0.415].

The estimated variance of the random intercept for participants was negligible (4.36 * 10^−14^), suggesting minimal inter-individual variability in baseline inter-event distances. These results indicate that intensity is a robust negative predictor of IOI, while sharpness and its interaction with intensity do not contribute significantly to explaining variability in distance.

### Peak modulation

**Figure 4.**
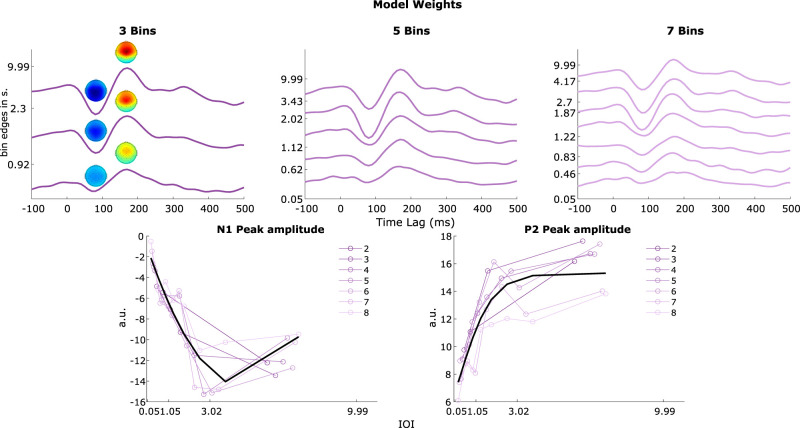
The top row shows model weights of three different bin models, i.e., 3, 5, and 7 bins. For the three-bin model, we also show the topographies at the N1 and P2 latencies for the different model weights. The *Y*-axis shows the upper edge of each bin. The bottom row shows the minimum and maximum magnitude of the N1 and P2 waves, respectively. The values are extracted for each bin of the seven different models. The models differ in their total number of bins, and bin edges vary to the uniform distribution constraint. On the bottom row, the left plot shows the distribution of N1 peak values as a function of bin edges. On the right, the same is displayed for the P2 values. The black line indicates the optimal model, fitted to all values.

Our results revealed amplitude modulation based on IOI. The larger the IOI, the larger the amplitude of the AEP. This finding was consistent across all variations of binning parameters. Neither the number of bins nor the specific constraints applied to the binning process significantly altered these findings. Specifically, we observed that the amplitude of the neural response was enhanced for sound events that followed a preceding sound at a greater IOI (Fig. 4). To quantify this effect, we extracted peak values of the N1 and P2 components from group-averaged TRFs. The results demonstrated a clear trend in which neural response amplitude increased as a function of IOI between tones. Notably, we found that greater temporal spacing elicited a larger N1 peak and a stronger P2 peak across all binning variations (Fig. 4).

To determine the relationship between the peak amplitudes and IOI, we fit a logistic, exponential, and polyfit model to the data. The first two models were inspired by existing literature (Zacharias et al., 2012; Herrmann et al., 2016). The results showed that the optimal model to describe N1 was the polyfit model of second degree (*R*^2^ = 0.84) and the logistic model for the P2 (*R*^2^ = 0.75).

**Figure 5.**
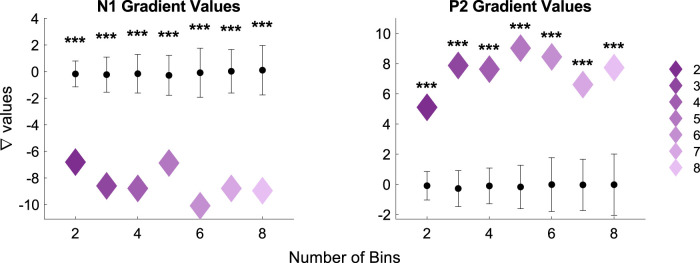
Displayed are the gradient amplitude values of the N1 and P2 for each binned model, respectively. The black bars indicate the standard deviation, and the dots the mean of the chance-level permutation values. The significance level here is indicated at *p < 0.05, **p < 0.01, and ***p < 0.000.

The results of the permutation testing revealed that the change of amplitude of the N1 and P2 for increasing IOI was significantly above the chance-level (*p* < 0.000; Fig. 5). This effect was found for all models.

### Prediction analysis

**Figure 6.**
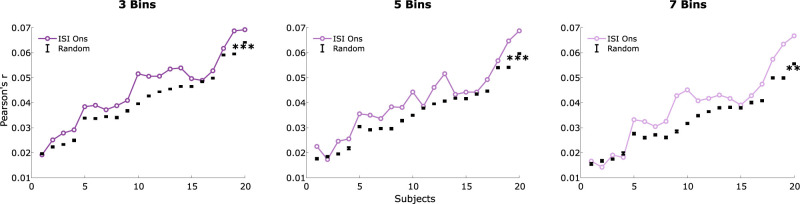
The plot shows the comparison of prediction accuracies between the binned models, i.e., 3, 5, and 7, and the random permutation model over participants. The significance level here is indicated at *p < 0.05, **p < 0.01, and ***p < 0.000.

Next, we examined whether incorporating IOI information into the neural model estimation improved the explained variability in the recorded signal. To do this, we compared the prediction accuracies of multiple models. First, we tested whether our IOI binned model outperformed chance-level predictions based on the results of the permutation testing (Fig. 6). The results showed that all models outperformed the random models significantly: (**2**: *W* = 202, *Z* = −3.62, *p* = 0.002, *ρ* = 0.57; **3**: *W* = 208, *Z* = −3.85, *p* = 0.001, *ρ* = 0.61; **4**: *W* = 204, *Z* = −3.7, *p* = 0.002, *ρ* = 0.58; **5**: *W* = 207, *Z* = −3.81, *p* = 0.001, *ρ* = 0.60; **6**: *W* = 210, *Z* = −3.92, *p* = 0.001, *ρ* = 0.62; **7**: *W* = 202, *Z* = −3.62, *p* = 0.002, *ρ* = 0.57; **8**: *W* = 198, *Z* = −3.47, *p* = 0.003, *ρ* = 0.55). The results indicated a non-linear relationship between model dimensionality and prediction accuracy. Specifically, the difference in prediction accuracy between structured and random models varied depending on the number of bins used. At extreme levels of model dimensionality, either very low or very high, the performance of the binned model only marginally exceeded the chance-level. In contrast, intermediate levels of dimensionality produced the most pronounced differences in prediction accuracy. The greatest improvement occurred when using three to six bins, suggesting an optimal balance between information preservation and model complexity.

#### Single model comparison

Following our comparison of the binned IOI models to the random models, we aimed to determine whether the inclusion of binned temporal information would outperform a simpler model based on a single binary onset vector. Specifically, we contrasted our more complex model to the single-vector case, which does not incorporate binning information.

**Figure 7.**
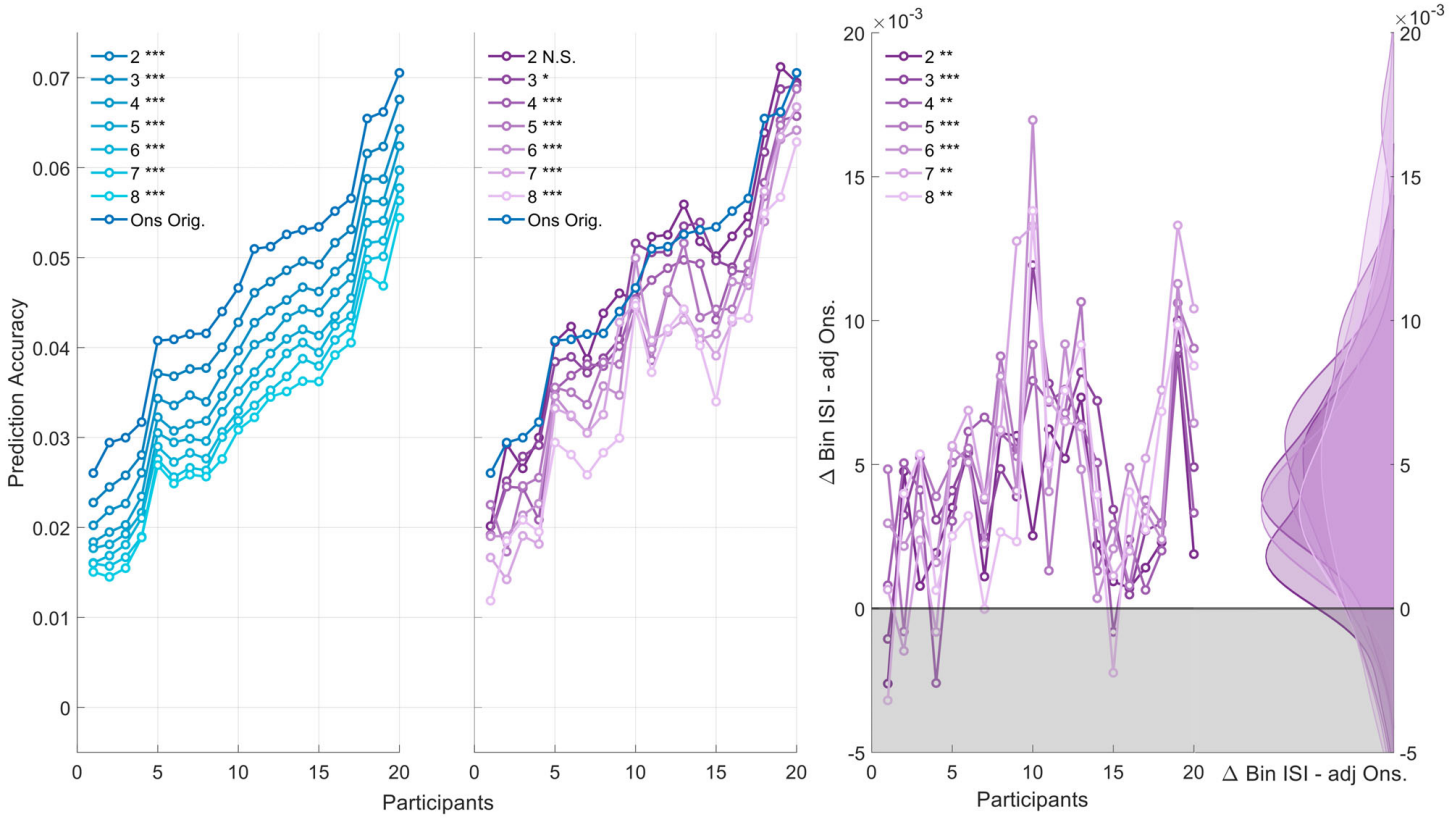
Shows the prediction accuracies over participants for different models. The left plot shows the prediction accuracies for the training adjusted onset model, which is based on the average number of onsets of the corresponding bin IOI model. The plot in the middle contrasts the prediction accuracy of the seven bin IOI models with the onset model. The plot on the right shows the difference between the bin IOI model and adjusted onset.

Despite observing amplitude modulation as a function of the temporal spacing of sound onsets, incorporating this information into model derivation did not yield higher prediction accuracies compared to using a single onset vector. In fact, the single onset model consistently outperformed the binned models across all cases (**3**: *W* = 176, *Z* = 2.65, *p* = 0.039, *ρ* = 0.42; **4**: *W* = 210, *Z* = 3.92, *p* = 0.001, *ρ* = 0.62; **5**: *W* = 210, *Z* = 3.92, *p* = 0.001, *ρ* = 0.62; **6**: *W* = 208, *Z* = 3.85, *p* = 0.001, *ρ* = 0.61; **7**: *W* = 210, *Z* = 3.92, *p* = 0.001, *ρ* = 0.62; **8**: *W* = 210, *Z* = 3.92, *p* = 0.001, *ρ* = 0.62). The only exception was the simplest binned model, which divided the data into two bins (**2**: *W* = 128, *Z* = 0.86, *p* = 1, *ρ* = 0.14; Fig. 7, middle). These results were stable regardless of whether binning was performed using linear or logarithmic spacing or when sample points per bin were uniformly distributed, as was the case here for the presented results. Additionally, accounting for condition differences did not significantly impact model performance.

Furthermore, we observed a deterioration of the prediction accuracy with an increasing number of dimensions. This suggests that data available for training may be a key factor driving the observed difference between the single and binned models. One potential concern is that the onset model and the binned IOI model may not be entirely comparable due to differences in the quantity of training data available for each set of feature weights.

To test whether, at comparable amounts of training data, our bin IOI model would capture neural data more optimally compared to the single onset model, we revisited the previous analysis. Specifically, we adjusted the number of onsets used for training in the single onset vector model to match the average number of onsets per bin for all binned models. For instance, in the case of the two-bin model, roughly 200 onsets per bin are available. Thus, we randomly selected 200 onsets in the single onset model for training. This process was repeated 100 times for each model, for each participant, and condition.

The results, shown in Figure 7, contrast the performance of the adjusted single onset models with the full single onset vector model. Trivially, every adjusted onset vector was outperformed by the full single onset vector (*W* = 210, *Z* = 3.92, *p* = 0.001, *ρ* = 0.62). Notably, the statistics hold for all comparisons, since parametric tests do not consider the mean difference between distributions. Here, the respective statistics of the effect size, *z* value, *W* rank, and *p* value represent the upper bound.

Given the impact of training data availability, we then revisited the comparison between the binned ISI models and the adjusted single onset vectors. The results indicate that the binned ISI models significantly outperformed their adjusted single onset counterparts across all bin variations (**2**: *W* = 199, *Z* = −3.51, *p* = 0.003, *ρ* = 0.78; **3**: *W* = 208, *Z* = −3.85, *p* = 0.001, *ρ* = 0.86; **4**: *W* = 201, *Z* = −3.58, *p* = 0.002, *ρ* = 0.80; **5**: *W* = 208, *Z* = −3.85, *p* = 0.001, *ρ* = 0.86; **6**: *W* = 210, *Z* = −3.92, *p* = 0.001, *ρ* = 0.88; **7**: *W* = 204, *Z* = −3.7, *p* = 0.002, *ρ* = 0.83; **8**: *W* = 198, *Z* = −3.47, *p* = 0.003, *ρ* = 0.78). These findings highlight the critical role of training data availability in the observed model performances for binary features.

#### Extended data analysis

When contrasting the prediction accuracy of the model containing both conditions with the single onset vector, we found that only the two-bin and three-bin models did not differ significantly from the single onset vector (**2**: *W* = 103, *Z* = −0.075, *p* = 1, *ρ* = 0.017; **3**: *W* = 142, *Z* = 1.38, *p* = 0.51, *ρ* = 0.31). For the more complex models (i.e., bin size >3), prediction accuracy was significantly lower compared to the single onset vector (**4**: *W* = 194, *Z* = −3.32, *p* = 0.005, *ρ* = 0.53; **5**: *W* = 190, *Z* = −3.17, *p* = 0.007, *ρ* = 0.50; **6**: *W* = 206, *Z* = −3.77, *p* = 0.001, *ρ* = 0.60; **7**: *W* = 204, *Z* = −3.7, *p* = 0.002, *ρ* = 0.58; **8**: *W* = 209, *Z* = −3.88, *p* = 0.001, *ρ* = 0.61).

**Figure 8.**
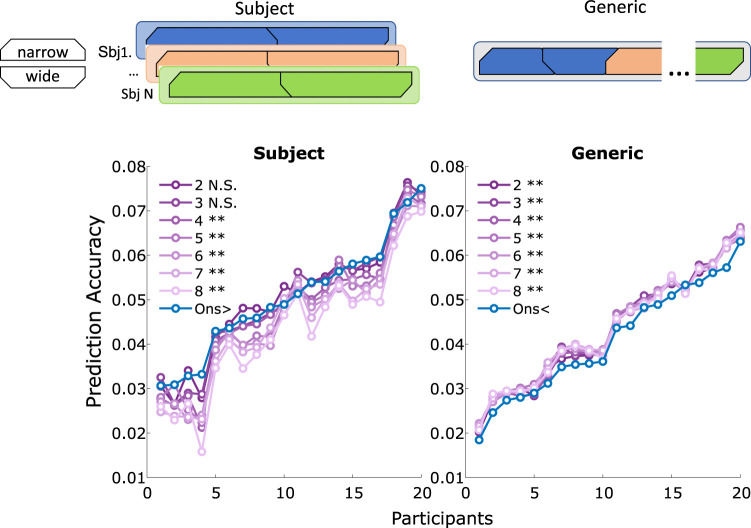
Shows the prediction accuracy of participants for different ways of training the data. The plot on the left shows the prediction accuracy over participants for merged condition data, where a model was trained on this longer dataset. The right side visualizes a generic model being trained. Here, each participant’s merged condition dataset served as a held-out test set once. The </ > next to the *Ons* model indicates the direction of significance. The significance level here is indicated at *p < 0.05, **p < 0.01, and ***p < 0.000.

We then trained a generic model using all available participant data, leaving one participant out as a held-out test set (Fig. 8). This was repeated for every participant once. The results showed that every binned model significantly outperformed the generic single onset model: (**2**: *W* = 3, *Z* = −3.81, *p* = 0.001, *ρ* = 0.60; **3**: *W* = 2, *Z* = −3.85, *p* = 0.001, *ρ* = 0.61; **4**: *W* = 8, *Z* = −3.62, *p* = 0.002, *ρ* = 0.57; **5**: *W* = 1, *Z* = −3.88, *p* = 0.001, *ρ* = 0.61; **6**: *W* = 4, *Z* = −3.77, *p* = 0.001, *ρ* = 0.60; **7**: *W* = 6, *Z* = −3.7, *p* = 0.002, *ρ* = 0.58; **8**: *W* = 7, *Z* = −3.66, *p* = 0.002, *ρ* = 0.58).

#### Curse of dimensionality

The relation between training data and training data required is known as the curse of dimensionality, where more complex models require exponentially more training data. One way to mitigate this issue is to parameterize the binary onset vector by the normalized range of distance values. This approach is similar to weighting word onsets in speech processing based on meta-information, such as surprisal. However, adding a parameterized version of the onset vector to the model did not improve prediction accuracy compared to the single onset model (*p* = 1).

## Discussion

Neural response attenuation to acoustic properties has been investigated mostly on isolated pure tones (Beauducel et al., 2000; Wang et al., 2008, 2022; May and Tiitinen, 2010; Herrmann et al., 2016). With this study, we extend those findings to naturalistic soundscapes.

This study provides evidence that neural attenuation is modulated by the IOI of sounds, generalizing validated lab findings to natural soundscapes. Here, the amplitude of the measured neural response was smallest when tones were close together and got larger the further tones were apart. This effect was robust over different numbers of pre-defined bins.

Based on these findings, we implemented the information into neural models to determine whether more neural variability could be explained. In summary, accounting for IOI information improves predictions of neural variability, provided that sufficient training data is available.

### Effects of IOI on neural data

Our results replicate classic attenuation effects observed for tone sequences (e.g., click trains or tone bursts; Okamoto et al., 2004; Wang et al., 2008; Costa-Faidella et al., 2011; Zacharias et al., 2012; Lanting et al., 2013; Herrmann et al., 2016). Crucially, we extend these findings to naturalistic soundscapes, showing that neural responses remain sensitive to inter-event timing despite high variability in acoustic properties. This suggests that neural attenuation with IOI is a fundamental organizing principle in auditory processing.

Interestingly, this amplitude increase appears to plateau for IOIs exceeding 3 s and for more complex models in the case of the N1 peak to decrease again. Beyond these values, additional increases in IOI no longer resulted in further amplitude increase. However, it is important to interpret this plateau cautiously, since specific IOIs were not explicitly manipulated but emerged as a consequence of the binning constraint. Furthermore, due to the naturalistic nature of our stimuli, direct comparisons with studies using strictly controlled IOI intervals are limited. As a result, we cannot draw precise conclusions about the exact time point at which this asymptote occurs. Additionally, separate functions best model the N1 and P2 amplitude values, respectively. This suggests that different mechanisms of attenuation underlie the N1 and P2 and thus possibly reflect separate neural generators (Altmann et al., 2008; Herrmann et al., 2016; López-Caballero et al., 2023).

### Neural mechanisms

Neural attenuation is a fundamental principle of sensory processing, observed across all sensory modalities and at multiple levels of the neural hierarchy, from peripheral receptors to cortical areas. This general phenomenon allows organisms to remain sensitive to new and changing stimuli by dynamically adjusting neural responsiveness in the face of repeated or sustained input (Ulanovsky et al., 2003; Dean et al., 2005; Hicks and McDermott, 2024).

Previous research has proposed two primary frameworks to explain auditory neural attenuation: habituation (often framed within a predictive coding framework) and adaptation. Habituation accounts argue that repeated or predictable stimuli generate sensory expectations, leading to reduced neural responses when those predictions are confirmed and increased responses when they are violated (Näätänen and Picton, 1987; Wang et al., 2008; Costa-Faidella et al., 2011; Silva et al., 2017; Ruusuvirta, 2021). In contrast, adaptation accounts propose a physiological explanation, suggesting that repeated stimulation leads to reduced neuronal responsiveness due to mechanisms such as synaptic fatigue or depletion, independent of stimulus predictability (Budd et al., 1998; May and Tiitinen, 2010; Rosburg and Mager, 2021; Rosburg et al., 2022; López-Caballero et al., 2023; López-Caballero, 2025).

Our study provides a unique opportunity to discuss these accounts because the naturalistic soundscape we used was highly variable spectrally and temporally. This random nature rules out the formation of stable sensory predictions, making a habituation or predictive coding explanation less likely. Additionally, the soundscape’s broad spectral variability suggests that the attenuation effects we observe are not simply the result of adaptation confined to narrowly tuned, organized auditory neurons. Interestingly, research suggests that temporal and spectral characteristics are adapted independently (Briley and Krumbholz, 2013). Thus, there may be separate neural processes underlying spectral and temporal adaptation.

In line, our findings point toward a general temporal sensitivity in auditory processing. We propose that the observed attenuation reflects broader adaptation mechanisms, such as synaptic depression or slow hyperpolarization, that operate independently of spectral content and are consistent with temporal recovery models described in previous work. The optimal parameters to describe the decay/recovery and exact models to depict the attenuation are still under debate (Wang et al., 2008; Zacharias et al., 2012; Lanting et al., 2013; Herrmann et al., 2016; Regev et al., 2021).

A promising neural substrate for these effects is the extralemniscal auditory pathway, which has been linked to stimulus-specific adaptation, detection of sudden environmental changes, and supramodal modulation of global brain states (Carbajal and Malmierca, 2018; Somervail et al., 2021; Shine et al., 2023; Willmore and King, 2023; Somervail et al., 2025). Importantly, it lacks the strict tonotopic organization of the lemniscal pathway and has been proposed as the neural substrate of the mismatch negativity, reflecting an error or novelty signal. Given its broader tuning and role in orienting responses, the extralemniscal system may underlie the non-spectral, time-sensitive attenuation we observed when sound events occurred in close temporal succession.

Despite our promising results, it remains an ongoing challenge to determine the underlying neural mechanisms that are responsible for neural attenuation. Although some theories have been proposed, it remains to be shown whether their integration explains attenuation in response to real-world soundscapes. Future studies should continue to systematically vary both spectral and temporal aspects jointly to determine whether the same or different mechanisms underlie the neural attenuation.

Although unclear where the attenuation occurs, our findings suggest that the temporal sensitivity of the auditory system persists even in complex, unpredictable environments. Importantly, they highlight that attenuation processes extend beyond stimulus-specific mechanisms in the face of dynamic sensory input.

### Potential confounding factors

Given the complexity of the soundscape under investigation, we examined whether systematic acoustic differences existed between sound events as a function of their IOI. Specifically, we focused on two features previously implicated in modulating neural response amplitudes (Drennan and Lalor, 2019; López-Caballero et al., 2023)—sound intensity and envelope sharpness.

Correlational analyses and linear mixed-effects modeling revealed a systematic difference in intensity with IOI, where sound events occurring in close succession tended to have a higher intensity than those spaced further apart. This relationship might be partially biased by the adaptive threshold to select onsets. Here, novelty peaks need to surpass a threshold that is based on the context of the soundscape. Thus, successive onsets may need a larger amplitude to exceed the context-driven threshold, driving the negative relationship between IOI and intensity.

Given the well-established association between increased stimulus intensity and stronger neural responses (Adler and Adler, 1989; Beauducel et al., 2000; Drennan and Lalor, 2019; López-Caballero et al., 2023), the fact that closely spaced events were more intense should have amplified rather than diminished their evoked responses. However, our results show the opposite effect: sounds occurring with shorter IOIs elicited attenuated neural responses. This dissociation suggests that intensity differences did not drive the observed IOI-related amplitude modulation, which is in line with findings of López-Caballero et al. (2023), who also found a dissociation between these two factors. On the contrary, intensity-related enhancement may have masked part of the IOI effect, making our findings a conservative estimate of the actual modulation associated with IOI.

Besides accounting for the sharpness and intensity, the creation of the soundscape itself may have introduced a bias, specifically, through the RMS normalization of every sound to the average level. While normalization is standard practice to control for loudness-related confounds in neural analyses, this procedure may somewhat reduce ecological validity by artificially equalizing loudness levels that naturally vary. Consequently, participants’ subjective perceptions and neural responses could have been slightly affected. However, given that individual sounds were individually adjusted prior to spatial separation using gain parameters (Kayser et al., 2009), we aimed to retain as much auditory realism as possible. Future studies could explicitly assess the impact of such loudness normalization procedures on subjective naturalness and corresponding neural dynamics.

Finally, we acknowledge the possibility that systematic motor-related artifacts or attentional biases could have influenced our neural findings. Specifically, motor responses associated with following verbal instructions or reacting to relevant tones could potentially reduce neural amplitudes (e.g., N1/P2). Conversely, increased attention toward verbal instructions might systematically enhance neural amplitudes for those stimuli compared to less relevant background sounds, potentially confounding our observed effects of onset intervals. However, several factors mitigate these concerns in our experimental design: first, the condition-relevant tones were randomly embedded within the soundscape, minimizing any systematic temporal alignment between attention or motor responses and particular stimulus categories. Second, motor responses occurred significantly later than the neural responses analyzed (e.g., N1/P2), reducing the likelihood that motor execution systematically influenced these early neural signals. Additionally, to further control for motor artifacts, our EEG preprocessing explicitly identified and removed motor-related EEG activity via ICA, substantially reducing potential residual contamination. Lastly, because acoustic onsets were derived indiscriminately from the raw soundscape, systematic biases induced by increased attention to relevant speech compared to irrelevant background stimuli are unlikely to have influenced our findings.

### Unconsidered factors influencing peak amplitude modulation

Our analysis, focused primarily on the IOI as a key modulator of neural responses while accounting for sound intensity and sharpness. However, given the complexity of naturalistic soundscapes, other acoustic and contextual factors may have played a role, which were not systematically investigated in the present study.

One such factor is the duration of the preceding sound. Lanting et al. (2013) reported that longer adapter durations led to greater N1 suppression in a paired-click paradigm, highlighting duration as a potential modulator of adaptation. Although this effect was shown using simple tones, its role in complex soundscapes remains uncertain and warrants further investigation.

Contextual predictability is another critical factor. Previous work has demonstrated that neural attenuation depends on the stimulation history (Zacharias et al., 2012; Herrmann et al., 2016). Notably, reduced attenuation effects are found under random IOI conditions compared to highly predictive sequences. How the general context impacts neural attenuation in autocorrelated soundscapes needs to be investigated by future studies. Evidence from a recent behavioral study suggests that response adaptation to stationary soundscapes occurs faster compared to those with increased spectral variability (Hicks and McDermott, 2024).

Finally, spectral similarity of successive sounds and global soundscape statistics has also been implicated in response attenuation. Herrmann et al. (2013) found stronger adaptation for spectrally similar tones. However, studies using more complex stimuli (e.g., vowels, animal vocalizations) have not observed such effects (Altmann et al., 2008; Silva et al., 2017). Given the broadband nature of our stimuli, the influence of spectral similarity remains ambiguous and was not directly tested here.

Taken together, these findings highlight that while IOI is a critical factor in auditory attenuation, other acoustic dimensions such as duration, spectral content, and stimulus context can also influence peak amplitude modulation. Future work should aim to incorporate these variables into more comprehensive models to better disentangle their individual and interactive contributions to auditory processing in naturalistic environments. As such, it would provide insights into how the brain processes complex soundscapes with greater detail.

### Prediction accuracy

#### Model performance

We have shown that integrating IOI provides meaningful information, as shown by the comparison between those models with random onset-bin allocation. Since both models contain onsets at identical time points, but only one assigns onsets based on the IOI between successive sound events, while the other does so randomly, the increased accuracy in the informed model indicates that IOI serves as a meaningful feature for neural prediction.

#### Trainings data availability

When we compared the IOI model against a single onset predictor (i.e., the simple onset model), performance was worse. This result was unexpected, given that the previous analysis showed the model to contain meaningful information. These findings also contrast with the study by Drennan and Lalor (2019), who showed that deriving features that account for the non-linear response of the brain improves model estimation and consequently the amount of neural variability explained.

This discrepancy can be explained by reduced training data per predictor: dividing the onset vector into multiple IOI-based bins leads to fewer events per bin. This impairs the derivation of reliable weights. This observation is crucial in explaining the inferior performance of the bin IOI model to the simple onset model. Given that prediction accuracy is strongly influenced by the availability of training data (Desai et al., 2023; Mesik and Wojtczak, 2023). To verify this interpretation, we controlled for data availability by reducing the simple onset model to match the number of events per bin in the IOI model. Under these conditions, the IOI model outperformed the reduced simple model, confirming that IOI carries predictive value.

We further tested this by increasing the available training data, either by pooling conditions within subjects or by training generic models across participants. In both cases, the IOI model benefited from increased data, often surpassing the simple model. Interestingly, the overall performance of generic models was lower compared to the individual models, and the difference between models was no longer visible. The reduced performance in the generic model compared to subject-specific models is likely due to the latter model better capturing individual nuances. This is in line with previous research showing that when sufficient training data is present, generic models underperform compared to subject-specific models (Mirkovic et al., 2015). Beyond this point, the subject-specific model is superior. The lack of difference between the bin IOI models suggests a ceiling effect of training data, indicating that further data would not yield additional performance gains. This underscores the need to balance feature complexity with the amount of available training data to avoid compromising model performance.

While increasing data availability helped address model complexity, we also examined whether simplifying the model could yield similar results. This approach was inspired by speech processing research, where word onset models are supplemented with parametric word surprisal scores to incorporate additional meaningful information into neural response estimation (Brodbeck et al., 2018). Analogously, we weighted binary onsets by their respective distance to the previous onset. However, this approach did not yield significant improvements in prediction accuracy. Highlighting that the binned approach is capturing non-linear neural dynamics.

#### Practical implications

These findings highlight a key trade-off: while incorporating temporal context (e.g., IOI) improves neural response prediction, increased model complexity requires sufficient training data to avoid performance loss. While shorter lab-based recordings may not provide enough data for models to benefit meaningfully from IOI-based features, longer recordings, particularly those collected in real-world, non-laboratory settings, offer an opportunity to leverage temporal information effectively. As longitudinal, everyday-life recordings (Hölle et al., 2021; Hölle and Bleichner, 2023; Rosenkranz et al., 2024; Korte et al., 2025a,b) become increasingly available, incorporating temporal structure such as IOI may significantly enhance model performance and our ability to predict neural responses in complex, naturalistic contexts.

### Conclusion

Our results provide important insights into how the brain processes complex soundscapes in everyday-life. We showed that temporal structure, specifically, the timing between sound events, is a critical dimension that modulates neural responses, even in highly variable, naturalistic settings. By demonstrating that shorter IOIs attenuate auditory neural responses and that IOI-based models can outperform simpler onset models (when data availability allows), we highlight the importance of integrating temporal features into the study of auditory scene analysis. These findings lay the groundwork for future research linking neural processing of soundscapes to perceptual, cognitive, and behavioral outcomes, advancing our understanding of how the brain interprets and adapts to the acoustic complexity of the real-world.

## Synthesis

Reviewing Editor: Anne Keitel, University of Dundee Decisions are customarily a result of the Reviewing Editor and the peer reviewers coming together and discussing their recommendations until a consensus is reached. When revisions are invited, a fact-based synthesis statement explaining their decision and outlining what is needed to prepare a revision will be listed below. The following reviewer(s) agreed to reveal their identity: Fran López-Caballero.

The manuscript has been assessed by one reviewer and the editor. The assessments were overall positive, with comments that should be straightforward to implement. Most comments refer to additional information and clarifications, as the manuscript should be interpretable as a standalone document, not just in conjunction with the previous publication. Most importantly, please add sufficient information on the participant sample and task. One more serious concern is that of motor-related artifacts. We wondered whether there might even be systematic movement artifacts, or other artifacts of the task, that could systematically influence the results? One possibility would be that if attention was directed towards the task and/or motor execution at specific times, this might lead to decreased N1/P2 amplitudes, which would confound the effect of onset intervals. Conversely, the verbal instructions might overall have been more attended than surgery sounds (as they are relevant), so amplitudes might be higher for those snippets, which might also confound the influence of onset intervals (if instructions also had e.g. longer onset intervals than other background stimuli). This also ties in with reviewer comment nr 3 below. More information on the paradigm and data cleaning would be necessary to assess this. Please also explicitly address the concern of the concurrent task in the manuscript (methods and/or discussion, depending on whether the task could have influenced results in a systematic way).

Additional editor comments:

I wonder whether the title could be improved. Currently, "Neural response attenuation for shorter inter-onset intervals between sounds in a natural soundscape" leaves it unclear what "shorter" refers to (shorter than what?). This is entirely up to you, but it might be preferable to use a clear title.

Line 42: "it has been shown that the N1 amplitude is scales nonlinearly with ISI" -> is scaled / scales.

You can find the unabridged reviewer comments below. Please address each comment below and the editor's comments above in a point-by-point manner.

Reviewer #1

Advances the Field (Required)

This paper advances the field by demonstrating that auditory refractoriness effects, traditionally observed with simple artificial sounds, also robustly occur in response to complex, naturalistic auditory scenes. This bridges the gap between controlled lab findings and real-world listening, enhancing ecological validity in auditory neuroscience.

Comments to the Authors (Required)

In this study, the authors aim to extend the understanding of auditory refractoriness, a well-known reduction in amplitude of electrophysiological signals (e.g., auditory evoked potentials) when auditory stimuli occur in rapid succession. They aim to generalize this effect beyond traditional, highly controlled stimuli sequences by investigating neural responses to more ecologically valid, naturalistic auditory input.

Using temporal response function (TRF) models, the authors consistently found modulation of N1 and P2 peak amplitudes as a function of Inter-Onset Intervals (IOIs). Their results further indicate that neural response amplitudes plateau for IOIs exceeding approximately 3 seconds.

Overall, this is a well-conducted and thorough study with clear aims that are successfully achieved. The work represents a valuable contribution and is a good fit for eNeuro. I support acceptance pending minor revisions, detailed in the comments below:

1) Basic participant information such as sample size, mean age, and eligibility criteria should be explicitly provided (even if the dataset is previously published) to enhance reproducibility and context.

2) The description of the soundscape's temporal structure and the two "conditions" is somewhat unclear. Specifically, how the total duration of ~16 minutes per condition is attained from the reported number and length of snippets and tones is difficult to follow. A schematic timeline or figure (possibly adapted from Rosenkranz et al., 2023) illustrating the sequence and timing of vocal instructions, conversation snippets, tones, and environmental sounds across conditions would improve clarity.

3) It is unclear which sound categories were included in the onset detection and TRF analyses. Since the soundscape contains various types (speech, environmental sounds, tones), clarifying whether all were treated equally or if some (e.g., background noise or irrelevant speech) were excluded or weighted differently would strengthen the interpretability of the results.

4) Given that the task required participants to perform a complex visuomotor activity (3D Tetris), potential contamination of EEG signals by motor-related potentials is a concern. Were motor artifacts considered and removed during preprocessing (e.g., through ICA)? Clarification on how motor-related EEG components were handled would be helpful.

5) The authors state that all sounds were RMS-normalized to ensure consistent loudness. It would be worth discussing whether this normalization may have affected the naturalness of the soundscape and potentially influenced participants' perception or neural responses. I suggest including a brief consideration of this point in the Discussion section (e.g., section 4.3).

## Author Response

We thank the reviewers for their thorough consideration of the manuscript and their valuable comments. We are grateful for the constructive feedback, which has greatly helped us to improve the manuscript. We have addressed all issues raised and marked all corresponding changes in the text. Furthermore, we have added the concerns regarding movement artifacts and RMS normalization to the discussion in order to acknowledge the insights resulting from the reviewers' input. We are convinced that the manuscript has improved considerably as a result of these revisions.

Synthesis of Reviews:

Computational Neuroscience Model Code Accessibility Comments for Author (Required): n/a Synthesis Statement for Author (Required):

The manuscript has been assessed by one reviewer and the editor. The assessments were overall positive, with comments that should be straightforward to implement. Most comments refer to additional information and clarifications, as the manuscript should be interpretable as a standalone document, not just in conjunction with the previous publication. Most importantly, please add sufficient information on the participant sample and task.

We thank both the editor and reviewer for highlighting this shortcoming of the paper. Please refer to our detailed answer and correction in response to the reviewer below. We hope that we have addressed the issue appropriately.

One more serious concern is that of motor-related artifacts. We wondered whether there might even be systematic movement artifacts, or other artifacts of the task, that could systematically influence the results? One possibility would be that if attention was directed towards the task and/or motor execution at specific times, this might lead to decreased N1/P2 amplitudes, which would confound the effect of onset intervals. Conversely, the verbal instructions might overall have been more attended than surgery sounds (as they are relevant), so amplitudes might be higher for those snippets, which might also confound the influence of onset intervals (if instructions also had e.g. longer onset intervals than other background stimuli). This also ties in with reviewer comment nr 3 below. More information on the paradigm and data cleaning would be necessary to assess this. Please also explicitly address the concern of the concurrent task in the manuscript (methods and/or discussion, depending on whether the task could have influenced results in a systematic way).

The editor raises an important point that has not been addressed in the previous version of the manuscript. We have attempted to address the points both mentioned by the editor and reviewer, and believe that this has improved the current version of the manuscript tremendously. Also, please refer to our answer to the reviewer's comments below.

Additional editor comments:

I wonder whether the title could be improved. Currently, "Neural response attenuation for shorter inter-onset intervals between sounds in a natural soundscape" leaves it unclear what "shorter" refers to (shorter than what?). This is entirely up to you, but it might be preferable to use a clear title.

We agree with the reviewer that the title could be improved for clarity. The new title goes as follows:

Neural response attenuates with decreasing inter-onset intervals between sounds in a natural soundscape.

Line 42: "it has been shown that the N1 amplitude is scales nonlinearly with ISI" -> is scaled / scales.

We have corrected this and thank the reviewer for spotting this mistake.

You can find the unabridged reviewer comments below. Please address each comment below and the editor's comments above in a point-by-point manner. ###### Reviewer #1 Advances the Field (Required) This paper advances the field by demonstrating that auditory refractoriness effects, traditionally observed with simple artificial sounds, also robustly occur in response to complex, naturalistic auditory scenes. This bridges the gap between controlled lab findings and real-world listening, enhancing ecological validity in auditory neuroscience.

Comments to the Authors (Required) In this study, the authors aim to extend the understanding of auditory refractoriness, a well-known reduction in amplitude of electrophysiological signals (e.g., auditory evoked potentials) when auditory stimuli occur in rapid succession. They aim to generalize this effect beyond traditional, highly controlled stimuli sequences by investigating neural responses to more ecologically valid, naturalistic auditory input.

Using temporal response function (TRF) models, the authors consistently found modulation of N1 and P2 peak amplitudes as a function of Inter-Onset Intervals (IOIs). Their results further indicate that neural response amplitudes plateau for IOIs exceeding approximately 3 seconds.

Overall, this is a well-conducted and thorough study with clear aims that are successfully achieved. The work represents a valuable contribution and is a good fit for eNeuro. I support acceptance pending minor revisions, detailed in the comments below:

1) Basic participant information such as sample size, mean age, and eligibility criteria should be explicitly provided (even if the dataset is previously published) to enhance reproducibility and context.

Thank you for the suggestion, we have added the sample size information in the main text as such. We hope that this adds sufficient information regarding the sample:

Line 98: In this dataset, 22 healthy, right-handed adults (age range: 20-30; 6 males, 16 females) were recruited through an online announcement. All participants provided informed consent and received monetary compensation. The sample size was determined based on previous studies investigating similar neural markers in natural settings (Scanlon et al., 2019; Hölle et al., 2021). Eligibility criteria included normal or corrected-to-normal vision, self-reported normal hearing, absence of psychological or neurological conditions, right-handedness, and compliance with COVID-19 hygiene regulations in place at the time of data collection. Two participants were excluded from analysis: one due to poor EEG data quality and another for not following task instructions. Therefore, the final analyzed sample comprised 20 participants (14 females, 6 males).

2) The description of the soundscape's temporal structure and the two "conditions" is somewhat unclear. Specifically, how the total duration of ~16 minutes per condition is attained from the reported number and length of snippets and tones is difficult to follow. A schematic timeline or figure (possibly adapted from Rosenkranz et al., 2023) illustrating the sequence and timing of vocal instructions, conversation snippets, tones, and environmental sounds across conditions would improve clarity.

We agree with the reviewer that the current state of the task description is too brief and leads to unnecessary confusion. Furthermore, we noted a slight error of the approximate length of the experiment which is 18, rather than 16 minutes. We have adapted the task description as such and hope that this clarifies the experimental setup.:

Subsection task Line 114: The goal of the original study was to investigate attentional effects in a surgical workplace scenario. For this...

Line 120: Specifically, participants were instructed to respond either to a distinct alarm tone (narrow attentional scope) or a less distinct beep tone (wide attentional scope). Both tones occurred within each condition, with only the target instructions differing between conditions.

Subsection Soundscape Line 145: Both tones (alarm and beep) were always presented in both experimental conditions, differing only in which tone participants were instructed to respond to. The timing of tone presentations within the soundscape was randomized individually for each participant. When randomization resulted in the beep tone overlapping with other tones (e.g., beep and alarm, or beep and irrelevant sounds), the overlapping sounds were returned to the stimulus pool and presented again at a new randomized time. This was done to obtain 48 non-overlapping trials to unbias the following EEG analysis of the tones. Consequently, slight variations in total condition duration occurred across participants. However, each participant consistently received the full stimulus set: 48 vocal instructions (~2-3 s each), 48 conversation snippets (mean duration 3.5{plus minus}1.5 s each), and 144 total tone presentations (48 each: alarm [200 ms], beep [60 ms], irrelevant [200 ms]), embedded within continuous environmental background sounds. Including brief silent intervals between stimuli and background environmental sounds, this procedure yielded an average soundscape duration of approximately 18 minutes per condition, totaling around 36 minutes across both conditions. Although durations varied slightly due to randomization, these differences were marginal and not expected to introduce systematic effects on time-on-task analyses. For additional clarity, a schematic timeline illustrating stimulus sequencing and timing (adapted from Rosenkranz et al., 2023) has been added.

Figure 1. Illustration of the experimental soundscape presented binaurally via headphones (left and right channel shown separately). Light grey indicates continuous surgical background noise. Dark grey marks task-irrelevant sound events, including vocal instructions and irrelevant speech snippets. Orange indicates the alarm tone relevant in the narrow-attention condition, while dark green marks the beep tone relevant in the wide-attention condition. The circular schematics below each discrete stimulus illustrate their spatial positions, manipulated using head-related transfer functions. Adapted from Investigating the attentional focus to workplace-related soundscapes in a complex audio visual motor task using EEG by M. Rosenkranz, T. Cetin, V. N. Uslar, &M. G. Bleichner, 2023, Frontiers in Neuroergonomics, 3, Article 1062227 (https://doi.org/10.3389/fnrgo.2022.1062227). Licensed under CC BY.

3) It is unclear which sound categories were included in the onset detection and TRF analyses. Since the soundscape contains various types (speech, environmental sounds, tones), clarifying whether all were treated equally or if some (e.g., background noise or irrelevant speech) were excluded or weighted differently would strengthen the interpretability of the results.

We thank the reviewer for this important comment. The onset detection was performed on the raw audio signal, and no distinction was made between specific sound categories (e.g., vocal instructions, conversation snippets, tones, or background noise). All detected acoustic onsets were therefore treated equally and weighted identically in the subsequent Temporal Response Function (TRF) analyses. This approach was chosen intentionally, since we focused on the purely acoustic temporal structure, irrespective of semantic or categorical distinctions. This was done to examine whether the observed temporal effects are present under these ecologically valid conditions.

We agree that it would be highly interesting to examine whether different stimulus categories contribute differently to the neural responses. Such an analysis would require sufficiently long and balanced material for each category. We see this as a promising avenue for future work with extended soundscape recordings, where differences between sound types could be systematically investigated.

Subsubsection Features line 260: Given that onset detection was performed on the raw audio signal, no distinction was made between specific sound categories (vocal instructions, conversation snippets, tones, or background noise). Therefore, all detected acoustic onsets were treated equally and weighted identically in the subsequent Temporal Response Function (TRF) analyses. This approach intentionally disregards semantic or categorical aspects of the soundscape to focus purely on acoustic temporal structure. For a detailed discussion of how using purely acoustic rather than content-informed onsets impacts the estimation and interpretation of neural responses, see Haupt et al. (2025).

4) Given that the task required participants to perform a complex visuomotor activity (3D Tetris), potential contamination of EEG signals by motor-related potentials is a concern. Were motor artifacts considered and removed during preprocessing (e.g., through ICA)? Clarification on how motor-related EEG components were handled would be helpful.

We thank the reviewer for highlighting this important consideration. Indeed, motor-related artefacts are always a concern in more open or real-life EEG setups and require special attention. On the one hand, they may compromise overall data quality; on the other hand, they might induce spurious effects if the motor artefact is time-locked to the stimulus. Motor-related EEG artifacts were accounted for during preprocessing using Independent Component Analysis (ICA). Specifically, ICA was computed on combined data across both experimental conditions to robustly identify artifact components. ICA components representing motor artifacts (i.e., muscle-related potentials) were automatically detected and removed using the EEGLAB function pop_icaflag, with predefined thresholds tailored specifically to identify and exclude muscle-generated EEG activity. Thus, EEG signals contaminated by motor-related potentials associated with the visuomotor task (3D Tetris) were systematically identified and excluded, reducing their potential influence on subsequent neural analyses. We have tried to highlight this specifically in the section "Preprocessing of EEG Data".

Line 204: Here, the conservative rejection thresholds were chosen to account for the button presses throughout the conditions.

Apart from removing potential artefacts using the well-validated IClabel function, reaction times to the condition-relevant tones were on average 0.814s (alarm, narrow) and 0.81s (beep, wide) (as reported in the original study). These are well after the peaks of interest. It is thus unlikely that muscle artifacts affect neural response in the N1 and P2 amplitude time window. As for the vocal instructions, the required action would only occur after the instructions finished. Given that the manual response occurred after the neural response to the onset of speech, we do not see any systematic way which the motor response could affect our effects.

Nonetheless, we agree with both the reviewer and editor that potential influences of muscle artefacts cannot be completely removed for single-trial button presses. However, since the placement of task-relevant sounds is random, meaning they could occur at any time, we strongly believe that potential residual movement artifacts did not influence the IOI analysis detrimentally. Furthermore, the participants had to react to different sounds in different conditions, i.e. while some sounds required a motor response in one condition, the same sound did not require a motor response in the other condition. We have addressed this issue in the discussion section as follows:

Line 654: Finally, we acknowledge the possibility that systematic motor-related artifacts or attentional biases could have influenced our neural findings. Specifically, motor responses associated with following verbal instructions or reacting to relevant tones could potentially reduce neural amplitudes (e.g., N1/P2). Conversely, increased attention towards verbal instructions might systematically enhance neural amplitudes for those stimuli compared to less relevant background sounds, potentially confounding our observed effects of onset intervals. However, several factors mitigate these concerns in our experimental design: First, the condition-relevant tones were randomly embedded within the soundscape, minimizing any systematic temporal alignment between attention or motor responses and particular stimulus categories. Second, motor responses occurred significantly later than the neural responses analyzed (e.g., N1/P2), reducing the likelihood that motor execution systematically influenced these early neural signals. Additionally, to further control for motor artifacts, our EEG preprocessing explicitly identified and removed motor-related EEG activity via independent component analysis (ICA), substantially reducing potential residual contamination. Lastly, because acoustic onsets were derived indiscriminately from the raw soundscape, systematic biases induced by increased attention to relevant speech compared to irrelevant background stimuli are unlikely to have influenced our findings.

5) The authors state that all sounds were RMS-normalized to ensure consistent loudness. It would be worth discussing whether this normalization may have affected the naturalness of the soundscape and potentially influenced participants' perception or neural responses. I suggest including a brief consideration of this point in the Discussion section (e.g., section 4.3).

We thank the reviewer for pointing out this aspect, which is vital for immersion. We acknowledge that RMS normalization of auditory stimuli, implemented to ensure consistent loudness across stimuli, could potentially have influenced the perceived naturalness of the soundscape. Our aim in creating the soundscape was to preserve a realistic and immersive auditory environment that reflects the complexity of real-world listening situations, rather than to artificially equalize all sounds. We have included this point in the discussion as such.

Line 643: Besides accounting for the sharpness and intensity, the creation of the soundscape itself may have introduced a bias, specifically, through the RMS normalization of every sound to the average level. While normalization is standard practice to control for loudness-related confounds in neural analyses, this procedure may somewhat reduce ecological validity by artificially equalizing loudness levels that naturally vary. Consequently, participants' subjective perceptions and neural responses could have been slightly affected. However, given that individual sounds were individually adjusted prior to spatial separation using gain parameters (Kayser et al., 2009), we aimed to retain as much auditory realism as possible. Future studies could explicitly assess the impact of such loudness normalization procedures on subjective naturalness and corresponding neural dynamics.
